# Cycling: Health Benefits and Risks

**DOI:** 10.1289/ehp.1003227

**Published:** 2011-03

**Authors:** Luc Int Panis

**Affiliations:** VITO (Flemish Institute for Technological Research), Mol, Belgium, E-mail: luc.intpanis@vito.be

de Hartog et al. (2010) quantified the balance between physical activity and air pollution and accident risks of cycling and concluded that the benefits outweigh the risks by an order of magnitude. This is the most comprehensive and quantitative comparison to date, based on the published data available at the time. In the weeks after publication of the article, two new relevant studies were published; this illustrates that a scientific answer to this question is urgent from the societal perspective. In many places cyclists are perceived to have a higher exposure to air pollution and a higher accident risk. Do the new data tilt the balance between the risks and benefits of cycling?

de Hartog et al. (2010) used a ventilation rate that is twice as high for cyclists as for car drivers. In a recent study in Belgium (Int Panis et al. 2010), we found that both the ventilation rate and the tidal volume were increased and that minute ventilation was 4.3 times higher in cyclists compared with car passengers (similar to the ratio of metabolic rates). The difference can further be explained by differences in cycling speeds and lung deposition resulting in a dose that is up to 9 times higher in cyclists.

The life expectancy (LE) loss estimated from substituting this ratio into the calculation by de Hartog et al. (2010) may thus offset most of the expected LE gain. However, this is unlikely because some studies have observed an LE gain in the presence of air pollution (Andersen et al. 2000). To resolve this conflict, it is important to consider the implicit assumptions in the comparison.

First, the higher dose ratios apply only to situations without route choice, although cyclists prefer to avoid motorized traffic, which exposes them to lower concentrations (Zuurbier et al. 2009). Second, an LE loss calculation based on long-term studies assumes a linear relationship between the risk and the daily dose. Exposure to short, high bursts of traffic exhaust may be different from an exposure to the same dose over a longer period. Assuming a linear exposure response function leads to overestimation of the impact of peak exposures. Third, cyclists are not a random sample from the general population. Air pollution mortality is often associated with the elderly and individuals with cardiovascular problems, but most cyclists are neither old nor very likely to suffer from bad health. Also, LE loss calculations cannot distinguish between situations in which a few people suffer a high LE loss or those in which many people have a small loss (Rabl 2003). Cyclists are generally young and in excellent health and therefore less vulnerable, implying that the relative risk used by de Hartog et al. (2010) is too high for application to this specific population.

In addition, accidents remain an important cause for concern. Aertsens et al. (2010) recently estimated the cost of minor bicycle accidents at an astonishing 0.12€/km cycled. Including the more serious accidents in the equation would yield a cost that could easily offset the value of the LE benefit calculated by de Hartog et al. (2010).

If the higher LE observed in present day cyclists can be transferred to people now taking up cycling, the benefits will probably be higher than the risks. However, it will be crucial to demonstrate that cycling increases physical activity. Without increased physical activity there are only risks, but reducing those risks may yield larger benefits than anticipated.
